# Aztreonam is a novel chemical inducer that promotes *Agrobacteium* transformation and lateral root development in soybean

**DOI:** 10.3389/fmicb.2023.1257270

**Published:** 2023-08-24

**Authors:** M. Waqar Khan, Wenqi Yang, Ke Yu, Xuebin Zhang

**Affiliations:** State Key Laboratory of Crop Stress Adaptation and Improvement, Henan Joint International Laboratory for Crop Multi-Omics Research, School of Life Sciences, Henan University, Kaifeng, China

**Keywords:** soybean, aztreonam, agrobacterium transformation, flavonoids, UHPLC–MS/MS

## Abstract

Agrobacterium-mediated soybean transformation is the simplest method of gene transfer. However, the low transformation due to the intractable nature of soybean genotypes hinders this process. The use of biochemicals (acetosyringone, cinnamic acid, flavonoids, etc.) plays an important role in increasing soybean transformation. These biochemicals induce chemotaxis and virulence gene activation during the infection process. Here we identified a biochemical, aztreonam (a monobactam), for high agrobacterium-mediated transformation in soybean. The soybean explants from three genotypes were inoculated with *A. tumefaciens* (GV3101) harboring the pMDC32 vector containing *hpt or the GmUbi-35S-GUS vector containing the GUS gene* during two separate events. High transient GUS expression was obtained during cotyledon explant culture on MS media supplemented with 2.5 mg/L aztreonam. The aztreonam-treated explants showed high efficiency in transient and stable transformation as compared to the untreated control. The transformation of aztreonam-treated explants during seed imbibition resulted in an average of 21.1% as compared to 13.2% in control by using the pMDC32 vector and 28.5 and 20.7% while using the GUS gene cassette, respectively. Based on these findings, the metabolic analysis of the explant after aztreonam treatment was assessed. The high accumulation of flavonoids was identified during an untargeted metabolic analysis. The quantification results showed a significantly high accumulation of the four compounds, i.e., genistein, apigenin, naringenin, and genistin, in cotyledon explants after 18 hours of aztreonam treatment. Alongside this, aztreonam also had some surprising effects on root elongation and lateral root formation when compared to indole-3-butyric acid (IBA). Our findings were limited to soybeans. However, the discovery of aztreonam and its effect on triggering flavonoids could lead to the potential role of aztreonam in the agrobacterium-mediated transformation of different crops.

## 1. Introduction

The soybean’s transformation efficiency is low as compared to other crops when using different methods. Biolistic and agrobacterium-mediated transformations have both been used as genetic transformation techniques. The combined approach of both of these methods has also been established to achieve high transformation in soybean. However, a minor increase has been reported while getting a maximum of 9% transgenic soybean, however, a higher transient transformation of 22.5% was obtained while using moist filter paper instead of solid media co-cultivation (Paes de Melo et al., 2020; Pareddy et al., 2020; Wang Y. et al., 2022). Other bacterial strains have also been identified as capable of transfroamtion. As compared to Agrobacterium and LBA44044, a large number of transnfroamtion has been obtained employing these bacterial strains (Cho et al., 2022). Neverthless, the simplicity and cost-effectiveness of agrobacterium-mediated transformation make it superior to biolistic. In order to use agrobacterium-mediated transformation, a high-efficiency transformation technique is still indispensable in soybeans and other plants (Xu et al., 2022). The application of various strategies, such as the use of biochemicals during bacterial infection or other physical tools and techniques that could speed transformation, has led to some increase in this process (Li et al., 2017). The use of phenolics, amino acids, flavonoids, and some surfactants greatly affects the transfroamtion efficiency of some plant species (Opabode, 2006; Subramoni et al., 2014). The amino acids L-glutamine and L-asparagine have positive impacts on soybean transformation and also exhibit some additional benefits (Chen et al., 2018). Acetosyringone, a phenolic substance, is commonly used to increase agrobacterium transformation. In addition, other phenolics have also been investigated for their potential role in agrobacterium transformation. Such a novel phenolic compound, fipexide, was investigated for shoot regeneration, callus formation, and *Agrobacterium* transformation in soybeans and some other plants (Nakano et al., 2018). Additionally, the naturally occurring phenolics (flavonoids) are crucial for the Agrobacterium-mediated transformation of a variety of plants (Bhattacharya et al., 2010; Mathesius, 2018). Some of these flavonoids play an important role in scavenging ROS and activating *Vir* genes (Agati et al., 2012). The investigation of different flavonoids like myricotin-3-gelactoside, narcissin, rutin, and apigenin-7-glucoside found them to be virE locus inducers (Zerback et al., 1989). Moreover, flavonoids and isoflavonoid compounds, such as genistein, apigenin, quercetin, luteolin, and naringenin, have been found to be positive inducers of emrAB operons. These operons synthesize the *Agrobacterium* inner and outer membrane fusion proteins. Among these flavonoids, quercetin and genistein were found to be more effective (Khemthong et al., 2019). The luteolin accumulation during seed imbibition leads to nodulation in alfalfa by *Rhizobium meliloti* (Hartwig and Phillips, 1991). Flavonoid have also been identified as an excellent antioxidant, as oxidative stress is the basic limiting factor in agrobacterium-mediated transformation (Agati et al., 2012).

During agrobacterium transformation, some plant hormones also play an important role. When used during the co-cultivation period, several auxins have been proven to positively stimulate transformation (Peña et al., 2004). The presence of gibberellic acid, zeatin ribosides, and myo-inositol during co-cultivation was found to be a positive regulator of transformation efficiency, seed germination, and plant growth (Biffen and Hanke, 1990; Olhoft et al., 2001; Jia et al., 2015). Some hormones, such as melatonin and sodium nitroprusside (a nitric oxide donor), were also discovered to be useful in addition to these substances. Melatonin reduced oxidative stress, leading to an increase in stable transformation in soybeans (Dan et al., 2015; Karthik et al., 2020).

Here we identified the novel secondary role of the antibiotic aztreonam, which belongs to the group monobactam, in increasing soybean transformation in this instance. Additionally, we evaluated its impact on flavonoid accumulation. Aztreonam was exclusively used to control bacterial infections in humans (Barradell and Brogden, 1992). The prospective use of the drug in plants has not, however, been supported by any research.

## 2. Materials and methods

### 2.1. Vectors assembly and transgene identification

For GUS and hygromycin gene transformation in soybean, two different vectors were used. A binary vectors, pMDC32, and GmUbi-3XFlag-35S-GUS/GFP were used to transfer *hpt* andGUS gene assemblies into the soybean genome, respectively. The CaMV-35S promoters were used to regulate the hygromycin phosphotransferase (*hpt*) and GUS genes (Supplementary Figures S1A,B). The pMDC32 vector assembly was transformed into *Agrobacterium tumefaciens* (GV3101) by the freeze–thaw method.

For the cloning of the GUS gene in the GmUbi-3XFlag vector, *in silico* vector assembly was performed in Snapgene software. The GUS gene was cloned into the AscI and XbaI restriction sites of the GmUbi-3XFlag-GFP vector. We additionally employed the CaMV-35S promoter in place of the GmUbi promoter for stable GUS expression. Restriction of the vector was carried out in a 50 mL aqueous reaction volume containing 1 μL AscI and 1 μL XbaI endonucleases (Takara), 1X Quick cut buffer (Takara), and 1 μg vector. The reaction mixture was incubated at 35°C for 2 h. The GUS gene was amplified from the pCambi 1,300 vector by using a pair of overlapping primers (Supplementary Table S1). Ligation of the destination vector and GUS gene was carried out in a 10 μL reaction volume using a one-step PCR cloning kit (Novoprotein) by following the user manual’s instructions. The true ligated GmUbi-35S-sGFP:GUS cassette was transformed into *A. tumefaciens* (GV3101) after colony PCR and sequence confirmation.

### 2.2. Seed handling and *Agrobacterium* inoculations

For half-cotyledonary node transformation and direct shoot organogenesis, freshly harvested soybean seeds from three soybean genotypes (Wm82, ZX-16, and ZX-3) were employed. The seeds were sterilized in 70% ethanol for 5 min and then in a 4.5% sodium hypochlorite solution for 15 min. After sterilization, the seeds were rinsed three times with ddH2O. The sterilized seeds were placed on germination medium containing quarter-strength MS medium with B5 vitamins, MgCl_2_.6H_2_O 0.02 mg/L, agar 0.7%, sucrose 1%, and aztreonam 2.5 mg/L, pH 5.7, as treatment and control without aztreonam. The seeds were kept in a growth chamber for 18 h (in the dark) at a constant temperature of 25°C and 70% relative humidity. During each event, the *Agrobacterium* containing the pMDC32 or GmUbi-3xFLAG-GUS assembly vectors was transformed into a soybean cotyledonary node. Prior to inoculation, *A. tumefaciens* was incubated overnight in YEP media containing selective antibiotics, as described by Olhoft et al. (2003). The *Agrobacterium* culture was centrifuged at 4000 rpm for 10 min. The supernatant was discarded, and the pellet was re-suspended in inoculation medium containing half-strength MS media with vitamins and 30 g/L sucrose, pH 5.4. The medium’s temperature was maintained at 21°C, and the OD was adjusted to 0.7 ± 0.02 at 600 nm. Following OD adjustment, 250 mg/L of L-cysteine and 200 μM acetosyringone were added to the inoculation medium. Before infiltration into the cotyledons, the inoculation medium was incubated at room temperature for 30 min. Imbibed seeds were divided into halves with care to ensure each cotyledon received a portion of the embryonic axis. The plumules were gently cut off. Multiple cuts were made adjacent to the cotyledonary node. According to Pareddy et al. (2020), the seeds were submerged in the inoculation mixture for at least 30 min while being shaken periodically.

### 2.3. Co-cultivation of explants

The half-cotyledonary explants were co-cultivated in 14 cm culture plates. Adaxially facing down, treated cotyledons were placed on sterile filter paper. The filter paper was soaked with liquid co-cultivation media (1/2 MS media with B5 vitamins, sucrose 2%, 6-BA 1.3 mg/L, acetosyringone 200 μM, L-Cysteine 250 mg/L, GA_3_ 0.3 mg/L, and aztreonam 2.5 mg/L (only in the case to treat explants) pH 5.4). However, the control media omitted aztreonam. The parafilms were wrapped tightly around the co-cultivation plates, which were then left in the dark for 4 days (under the aforementioned germination conditions). In order to eradicate the adhering *Agrobacterium*, the cotyledons were briefly washed in washing medium (as indicated for co-cultivation supplemented with 250 mg/L carbenicillin and 250 mg/L cefotaxime) for 30 min. Following this, the explants were transferred to shoot induction media.

### 2.4. Shoot induction media (SIM) and explant growth

For shoot organogenesis, we used our newly optimized SIM (Full-strength MS with B5 vitamins, MgCl_2._ 6H_2_O 0.2 mg/L, agar 0.7%, 6-BA 1.3 mg/L (Solarbio IB0100), spermidine 2.5 mg/L (Solarbio cat# S8030), N6-(2-isopentenyl) adenine 2iP 0.4 mg/L (cat# B24576), kinetin 0.3 mg/L (Solarbio, cat# K8011), GA_3_ 0.3 mg/L (Solarbio cat# G8910), cefotaxime 150 mg/L, carbenicillin 200 mg/L (Solarbio), 2-(N-Morpholino) ethane sulfonic acid monohydrate (MES) (Sigma Aldrich pcode 102,461,002) 1.5 g/L, pH 5.7). Cotyledonary explants were placed 45 degrees apart horizontally in SIM. The explants were cultured in a growth chamber under fully controlled conditions (25 ± 1°C, 60% white light intensities, and 70% humidity) for 16 h of light and 8 h of darkness. After 14 days in each sub-culture, the explants were transferred to new SIM media. When necessary for the aforementioned various experiments, elongating shoots (2–3 cm) were cut off from the cotyledon explant after 14 days. The excised shoots were rooted on root induction media containing half-strength MS with B5 vitamins, 2% sucrose, 0.75 mg/L MgCl2. 6H2O, 1.5 g/L MES, 4 mg/L IBA, 150 mg/L cefotaxime, and 180 mg/L carbenicillin, pH 5.7. Instead of IBA, some explants were planted on RIM supplemented with 2.5 mg/L aztreonam. The rooted seedlings were transferred to 6 cm^2^ pots containing a vermiculite:peat (1:1) mix. The trays containing pots were covered with transparent plastic humidity domes. After 7 days of acclimatization, the seedlings were transferred to the greenhouse in large (14 cm) pots.

### 2.5. Transgene identification

In the greenhouse, the fully acclimatized seedlings were allowed to grow and mature. With some modest modifications, the CTAB technique was used to extract the total genomic DNA from soybean lines (Cullings, 1992). The PCR products were resolved on a 2% agarose gel. After being transfromed with *Agrobacterium* containing the pMDC32 vector, the cotyledonary explants were initially evaluated on MS medium supplemented with hygromycin B. During the first, second, and third shifts of explants to fresh medium, hygromycine B concentrations of 0, 5, and 10 mg/L were used, respectively. After hygromycin selection, the transgene that survived was planted on root induction medium (RIM). PCR was conducted for the identification of the *hpt* gene from soybean lines with a pair of *hpt*-specific primers. The forward and reverse primer sequences were *hpt*-forward (ATTTGTGTACGCCCGACAGT) and *hpt*-reverse (CTCTCGGAGGGCGAAGAATC). PCR cycle conditions were 94°C for 4 min, 94°C for 30 s, 51.3°C for 30 s, 72°C for 30 s (34 cycles), 72°C final extension for 5 min, and then 4°C hold. However, for T1 transgene identification, the number of PCR cycles was raised to 40. The PCR products were resolved on a 1.5% agarose gel. The vector and non-transformed soybean lines were used as positive and negative controls in the gel, respectively. The putative transgenic soybean lines’ positive bands at a length of 840 bp were identified as *hpt* gene bands. Following the directions in the user’s manual, the Omega Bio-TEK (D2500-02) gel extraction kit was used to extract the DNA bands from the agarose gel. Sequencing provided further assurance of the targeted band. At maturity, seeds from positive transgenic T0 plants were harvested. In the T1 generation, segregation in transgenic events was evaluated. Similar procedures were employed for the identification of transgenic lines, as mentioned before.

### 2.6. GUS histochemical analysis

Soybean explants from three genotypes were subjected to GUS histochemical analysis. After 4 days of co-cultivation, GUS expression was examined in the inoculated epicotyl sections. Regenerated shoots were then tested 14 days later. The GUS staining buffer (50 mM NaH2PO4 (pH 7.2), 10 mM Na2EDTA, 0.1% (v/v) Triton-X, 1 mM K4Fe(CN)6, 1 mM K3Fe(CN)6, and 2 mM X-gluc) was used to stain the inoculated cotyledon parts (Jefferson et al., 1987). The cotyledon explants were incubated in GUS staining buffer for 12 h at 37°C. The green parts were cleared of chlorophyll for 1 hour with 95% ethanol and then for 5 hours with 70% ethanol. The GUS-stained explants were visualized under a stereomicroscope (Olympus SZX7, Japan). Transient transformation was calculated on the basis of positive explants for both aztreonam-treated and control explants.

### 2.7. Aztreonam treatment and lateral root initiation

To determine the effect of aztreonam on soybean root. The newly excised explants were planted in 1/2 MS media with 1% sucrose, 0.7% agar, 100 mg/L carbenicillin, and 100 mg/L cefotaxime, with 2.5 mg/L aztreonam (treatment) and IBA as a control. Aztreonam and IBA were added after the media had been autoclaved. Aztreonam’s performance was assessed in a comparative study of root induction and elongation using indole-3-butyric acid (IBA) as a control. More than six explants were cultured in each RIM medium pot and allowed to root. After 7 days of culture, root length and the number of lateral roots were evaluated in treated and control explants.

### 2.8. Untargeted metabolic analysis of aztreonam treated explants

We conducted an untargeted metabolic study on soybean explants to find out the metabolic modulation following aztreonam application. The soybean seeds were cultured on solid MS medium containing 2.5 mg/L aztreonam (treated) and without aztreonam (control). Six seeds in each pot were given 18 h of regulated soaking at 25 degrees Celsius, 70% humidity, a 16/8 light/dark cycle, and 60% white florescence light. The epicotyl and cotyledon portions were collected in separate tubes. The cotyledons were washed with tap water to remove adherent MS media. The samples were quickly frozen in liquid nitrogen before being crushed into a fine powder. From each sample, 100 mg of powder was collected in 5 mL polyethylene tubes. The powder was dissolved in 3 mL of 80% methanol. The samples were homogenized (Heidolph Shaker and Mixer, Germany) for 10 min at 4000 rpm. Centrifugation of the homogenate was performed at 4000 rpm for 15 min. The supernatant from each sample was collected in a fresh 5 mL tube. The solution was desiccated (in an Eppendorf centrifuge and concentrator) for 6 h at 30°C. The dried masses were redissolved in 300 μL of an 80% cold methanol and 0.01% formic acid (v/v) solution. Homogenate was filtered using a syringe filter (0.2 μM, low protein binding) before filling an HPLC vial. Each HPLC vial was filled with 200 μL. To identify contaminants or analytical errors during sample processing, a field blank sample was employed. For quality control, an even volume of each sample was taken and mixed in a single HPLC vial. Chrysin (1 μL/mL) was used as an internal standard. The experiment was repeated three times with three biological replicates.

For the extraction of flavonoid compounds, 96% (v/v) ethanol was used. From the laboratory chemicals repository, five HPLC-grade standards (genistein, apigenin, naringenin, luteolin, and quercetin) were obtained. The compounds were dissolved in DMSO to create a standard solution for measuring flavonoids in soybean tissue. In order to generate calibration curves, standards were further diluted and run through a UPLC-QqQ-MS/MS apparatus.

### 2.9. UHPLC–MS/MS analysis

The metabolic analysis was conducted using two separate UHPLC systems. For an untargeted metabolic investigation, the samples, a control, and a blank were placed into the LC–MS Q-Executive hybrid quadrupole-orbitrap mass spectrometer (Thermo Scientific United States). The primary assembly was equipped with an auto-detector, auto-sampler, and pump that were all regularly monitored. The C18 column (hypersil gold 1.9UM 100×2.1 mm column, USA) was used for the separation of metabolites. Flavonoids were quantified using the ultra-high performance liquid chromatography triple quadrupole mass spectrometry technique (UHPLC-QqQ-MS/MS) (Acquity UPLC I-Class Plus). A binary solvent phase was used as an elution gradient system for LC–MS/MS. These solvents contained 0.1% formic acid/water (solvent A) and methanol (solvent B). Both solvents were degassed in a Branson Ultrasonic Cleaner USA (Model CPX5800H-C) for 10 min before use. The elution gradient was used as follows: 0 min 10% B; 2 min 10% B; 10 min 50% B; 10 min 80% B; 13 min 95% B; 14 min 95% B; 14 min 10% B; and 18 min 10% B. The total run time ranged from 0 to 18 min. The flow rate was 0.3 mL/min, and the sample injection volume was 10 μL. The wavelength ranged from 190 to 500 nm. The sheath gas flow rate of 35, the auxiliary gas flow rate of 15, and the sweep gas flow rate of 0 were maintained. Spray voltage and current were maintained at 3.21 kV and 16.40 angstrom, respectively. The auxiliary gas heater temperature was 348°C, while the capillary temperature was 320°C. The spectra were acquired in negative and positive ionization modes over a mass-to-charge (m/z) ratio ranging from 50 to 1,200. The data was collected at a rate of 0.2–250 Hz. The scan range for both positive and negative ionization modes was 70 to 1,050 m/z, with a full MS resolution of 70,000.

The binary solvents chose in the elution gradient technique for quantitative determination of flavonoids were (A) water/formic acid (0.01%) and (B) 80% methanol/0.01% formic acid. A stationary-phase Acquity UPLC Cortecs T3 column (2.1x100mm, 1.6um, P/N: 186008499) was used. For the elution gradient, 0.4 mL/min flow was maintained. The elution gradient was set as 0 min 5% B, 0.5 min 5% B, 2.5 min 20% B, 13 min 80% B, 15 min 95% B, 16 min 95% B, and 18 min 5% B. All the other conditions were the same as mentioned above.

### 2.10. Data sorting and retrieval

The accurate m/z value that was passed down from ESI-Q-TRAP LC–MS/MS was analyzed further. Fragmentation pattern and retention time were processed in thermo-scientific proteome discoverer software. The metabolite identification and structural similarities were retrieved by following the best match percentage rules, m/z fragmentation pattern, and least molecular weight differences<0.01. The compounds with m/z fragments that matched ≥70% with reference libraries were kept for further analysis. To remove the ambiguity, some identified compounds were further verified by the PubChem^1^ and MzCloud^2^ databases. The metabolic data were further sorted through MS-Excel and finalized for analysis by removing duplicate and ambiguous values.

### 2.11. Statistical analysis

Multiple-variate analysis of untargeted metabolites was performed using the web interface of the MetaboAnalyst V 5.0 companion with the R package (www.metaboanalyst.ca). The acquired data was normalized prior to analysis. For the analysis of statistical significance among groups, a one-way ANOVA was used. Multivariate strategies for comprehensive data analysis, like a supervised method (partial least squares discriminant analysis [PLSDA]) and an unsupervised method (hierarchical clustering with heat map), were used. Differentially accumulated compounds were identified through PLS-DA and a variable importance in projection (VIP) score using a significance threshold of *p* < 0.05. A post-hoc analysis of Fisher’s least significant difference was generated using the Pearson distance measure and the Ward clustering algorithm. For the assessment of variation among root length and number of lateral roots, an unpaired T-test was applied in GraphPad Prism 8 and MS Excel 2016. The pathway analysis and metabolite enrichment analysis were performed on the MetaboAnalyst platform by comparing sorted metabolites with *Arabidopsis* metabolite libraries. For graphics and figure illustrations, CorelDraw Graphic Suite 2021 was used. The Snapgene software (4.1.9 version) was used for computer simulations of vector assembly and gene cloning. All the experiments were repeated at least three times with three or more biological replicates.

## 3. Results

### 3.1. Aztreonam’s surprising effects on soybean transformation

**Figure 1:**
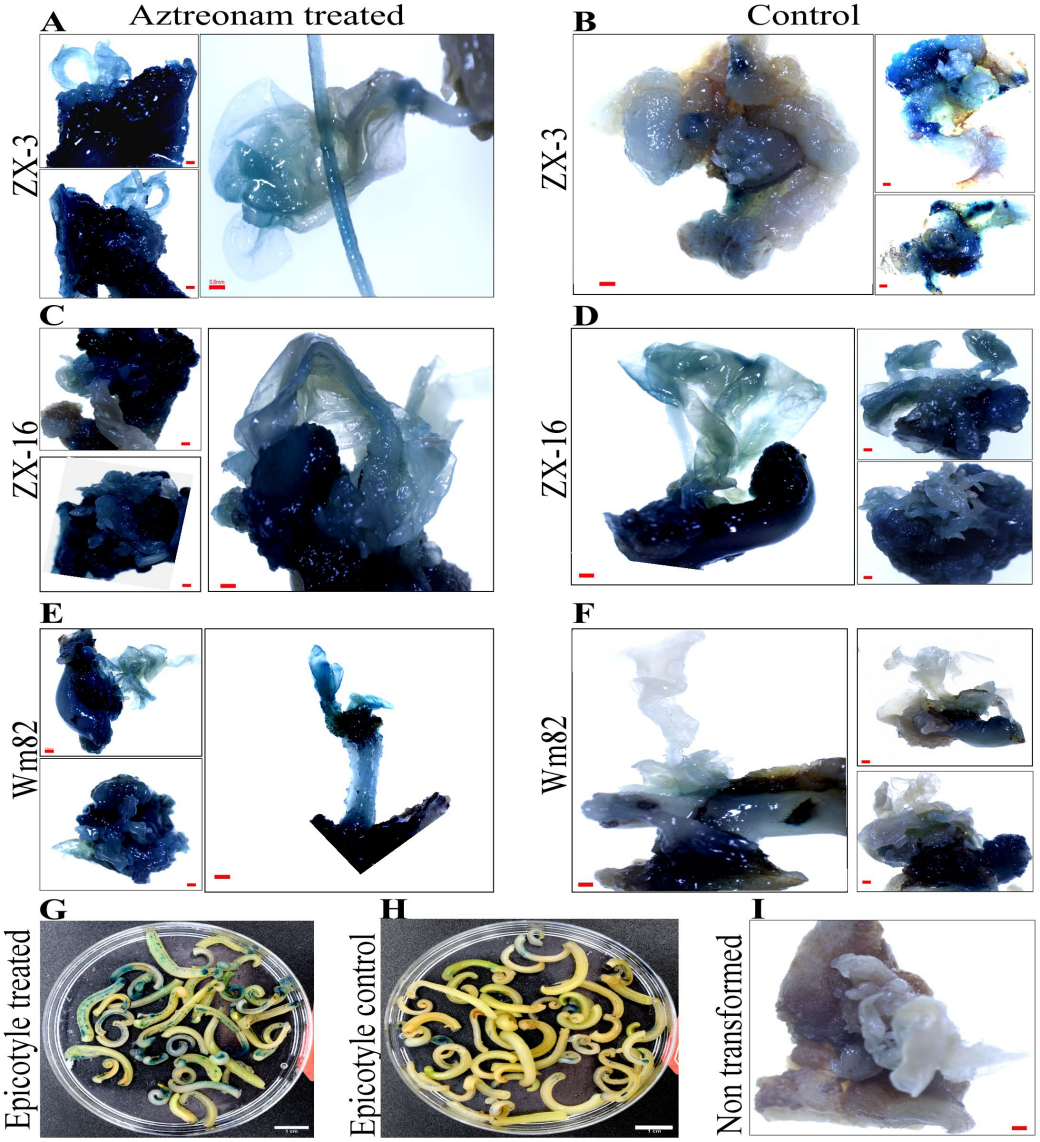
Positive GUS expression of aztreonam-treated and control explants of three soybean genotypes. Scale bars for panels **(A–F)** and **(I)** are 0.8 mm, and **(G)** and **(H)** are 1 cm. Panels **(A,C,E)** indicate the aztreonam-treated explants from three soybean genotypes, while **(B,D,F)** show the control for these genotypes. Panel **I** indicates the non-transformed control cotyledonary explant treated with GUS. Panels **(G,H)** show the epicotyl of aztreonam-treated and control explants stained with GUS after 4 days of co-cultivation.

Aztreonam is a synthetic antibiotic of the monobactam class that is typically used to treat severe blood, skin, stomach, lungs, and urinary tract infections in humans. The possible function of aztreonam in soybeans or other plant species has not been demonstrated. We chose aztreonam to assess its impact during soybean transformation based on the various biological characteristics of the other members of this class. After performing repeated tests, we discovered the remarkable effects of aztreonam on soybean seedlings. Initially, aztreonam was tested on sprouting soybean seedlings on MS media to determine whether it had adverse effects. Furthermore, we discovered the optimum concentration for effective agrobacterium transformation after successfully completing the initial trials. During an investigation, different concentrations of aztreonam were tested in MS media during the seed imbibition period. For soybean transformation, 2.5 mg/L was shown to be the optimal concentration. The soybean seeds were allowed for agrobacterium infiltration after 18 h of imbibition on MS media supplemented with 2.5 mg/L aztreonam. The same concentration of aztreonam was also used during the co-cultivation of the explants. No inhibitory effects of aztreonam on *Agrobacterium* were investigated at such a low concentration. The cotyledonary explants without aztreonam were kept as controls. When compared to the control, the elongated epicotyl segment of the aztreonam-treated explants showed stronger signals and a greater number of positive GUS-stained tissues (Figures 1G,H). The half-cotyledon explants were then subjected to shoot organogenesis on SIM. Our newly optimized shoot induction media produced fast shoot regeneration and growth (Supplementary Figure S2). After 14 days of culture, the half-cotyledon explants with shoot buds and callus were allowed a second round of GUS staining. The aztreonam-treated explants from all three soybean genotypes (Wm82, ZX-16, and ZX-3) showed high GUS expression (Figures 1A,C,E) as compared to their non-treated controls (Figures 1B,D,F) however, there was no GUS positive stain was identified in non-transformed control (Figure 1I). A significantly high number of GUS-positive stems were calculated in the aztreonam-treated explants. The complete GUS stained shoots were considered stable. The transformation efficiencies of the treated groups of individual genotypes were found to be high. However, we calculated the transformation efficiencies of all the genotypes collectively to increase confidence in the data. The combined average transformation for all genotypes after aztreonam treatment was 28.5%, compared to 20.7% in the control (Table 1).

**Table 1:** Transformation % using GUS histochemical analysis of different soybean genotypes.

Soybean genotypes		Total no of explants	No of GUS positive explants	GUS transient transformation %
Wm82	Aztreonam	70	20	28
ZX-3		80	30	37.5
ZX-16		50	10	20
**Total**		**200**	**60**	**28.5 Average**
Wm82	Control	50	10	20
ZX-3		80	23	28.7
ZX-16		60	8	13.3
**Total**		**190**	**41**	**20.7 average**

The bold values in table represent the cumulative numbers of all the above events.

**Figure 2:**
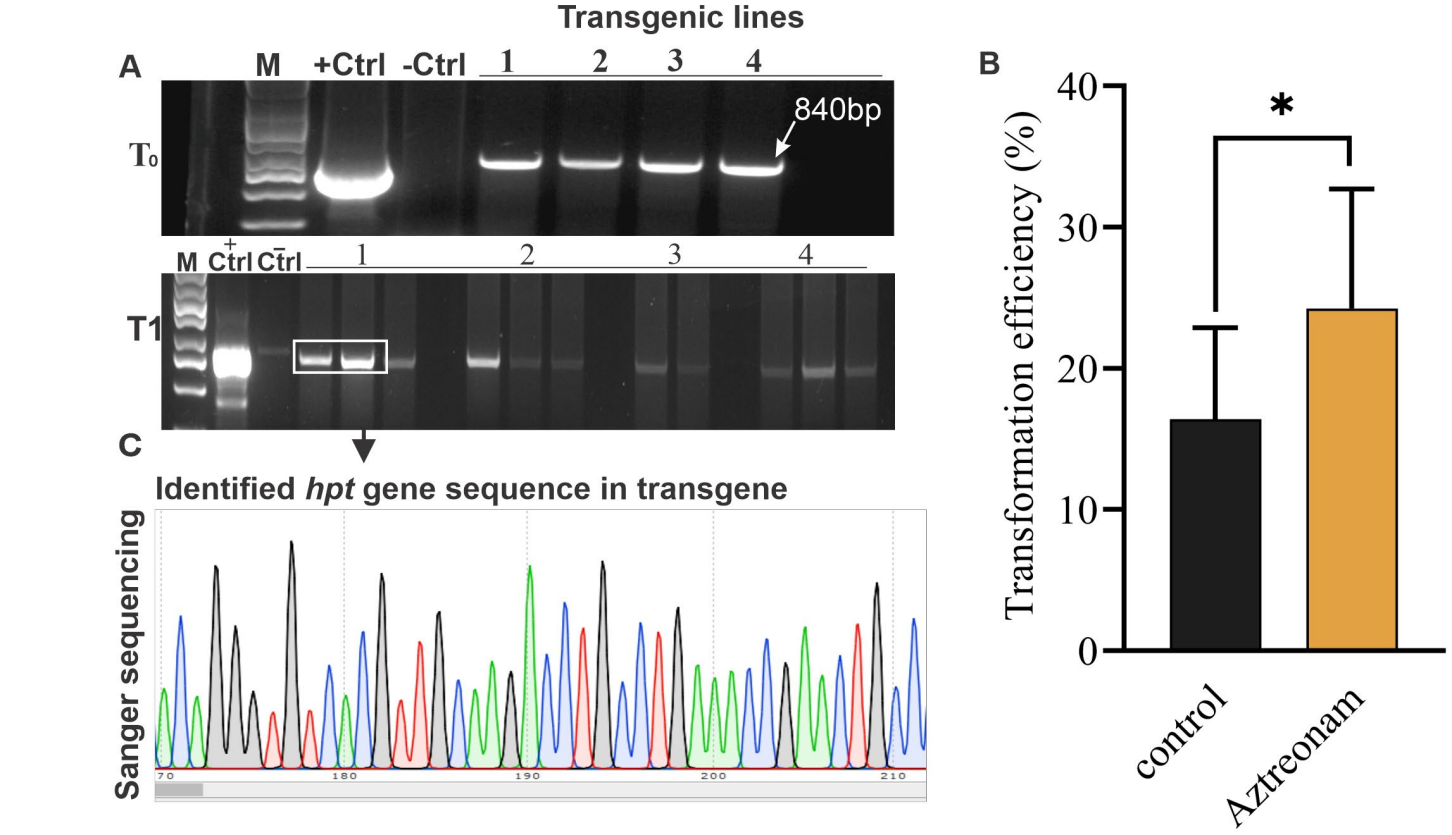
*hpt* gene-specific PCR and sequencing were used to identify putative transgenes. Panel **(A)** shows the PCR products resolved on a 1.5% agarose gel. Each line in the gel represents each event. The gel indicates both the results of the T1 and T2 transgenes. The first line is the DNA marker, the second is vector control (+Ctrl), the third is non-transformed soybean control (-Ctrl), and the lines numbered from 1 to 4 indicate each transgenic line. The identified band length is 840 bp. Graph **(B)** shows the transformation efficiency of the aztreonam-treated explants. Error bars represent 1 ± SD from the mean. While the *T*-test showed significant differences in the value between groups at the 0.05 level, Panel **(C)** shows the sequence results of the PCR product for hpt gene identification in soybean transgenic lines.

However, positive plants for the *hpt* transgene were initially selected using MS medium supplemented with hygromycin B. Identification of the positive seedlings was carried out through PCR. The *hpt* gene fragment was identified in most of the positive transgenic lines (Figure 2A). The *hpt* gene-specific sequences in these transformed soybean lines were validated by sequencing the amplified fragment (Figure 2C). The transformation efficiency of the treated and control seedlings was calculated based on positive events. The highest average transformation efficiency of control explants was 13.1%, while the average transformation efficiency of explants treated with aztreonam was 21.1% (Figure 2B and Table 2). The average transformation of the aztreonam-treated explants was higher during both events compared to the control. The T1 generation was raised after harvesting the T0 transgenic seeds. Since we were only able to obtain *hpt* transgenic seeds, Mendelian co-segregation of alleles was evident in the T1 generation. Similar procedures were employed for the identification of Transgenic during T1 generation, as mentioned for T0 Transgenic lines identifications. However, the seeds from T1 transgenic lines were grown on hygromycin B supplemented media for selection of positive transgenic lines (Supplementary Figure S4).

**Table 2:** Transformation percentage of soybean of William28 treated with aztreonam and control.

Treatment	Transformation events with vector type	Total no of ex-plants inoculated	Ex-plant survived	Positive transgene	Transformation efficiency
Aztreonam	pMDC32	360	225	72	21.1% average
Control	pMDC32	360	205	46	13.2% average

The *hpt* gene was used for the identification of transgenic lines. Transformation efficiency = Number positive explants/total no of inoculated explants multiplied by 100.

### 3.2. Aztreonam promotes root development in soybean

**Figure 3:**
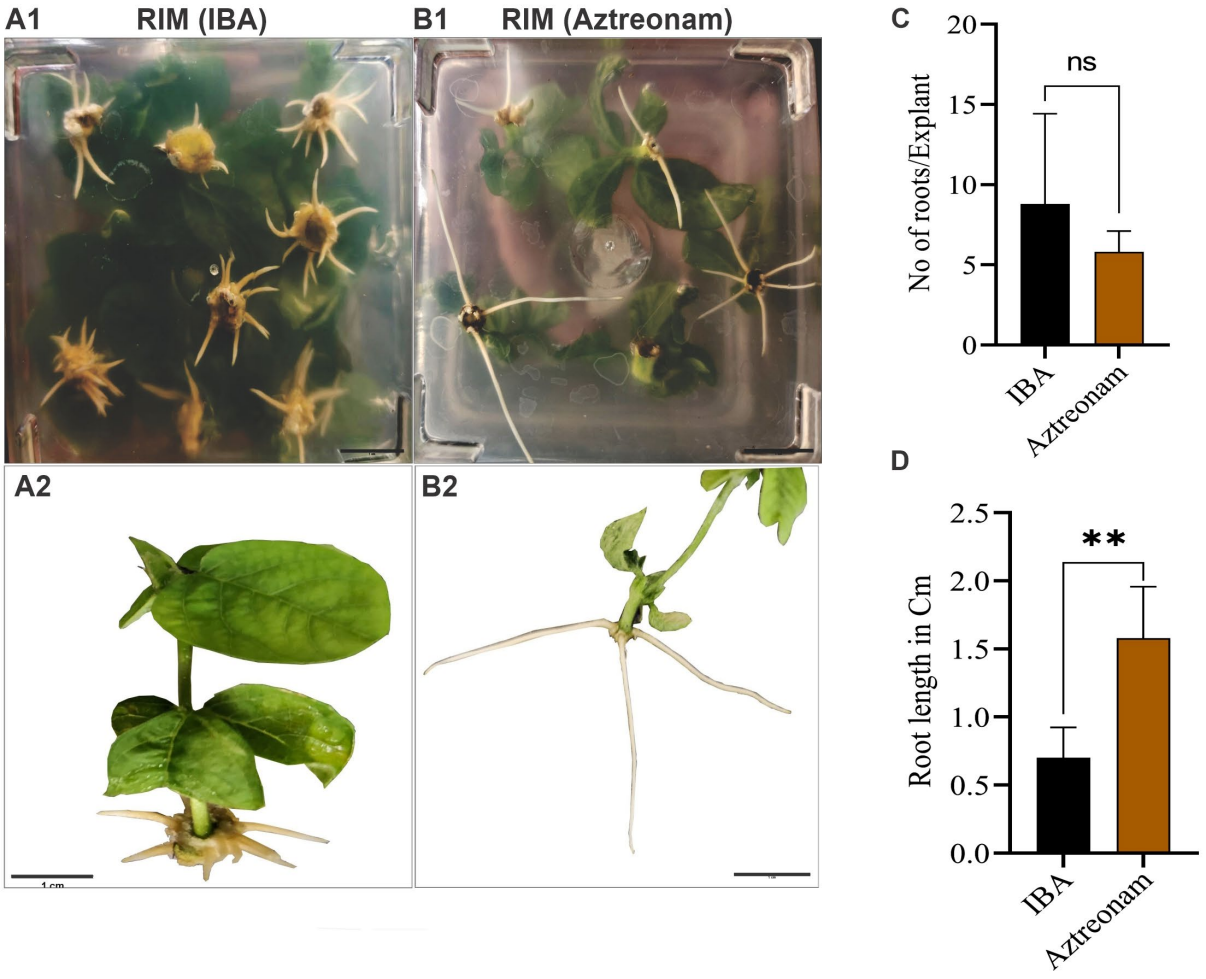
Aztreonam’s effect on root morphology. Panels **(A1,B1)** show the soybean shoots on root induction media (RIM) supplemented with IBA **(A1)** and aztreonam **(B1)**. Panels **(A2,B2)** indicate individual seedlings after IBA and aztreonam treatments, respectively. Graph **(C)** shows the comparison of IBA and aztreonam in the number of roots. The *F*-test statistic was applied to show the significance of the data at *p* < 0.05. The graph **(D)** indicates the comparison of root length. Error bars for both graphs show SD from the mean value. Scale bar = 1 cm.

We explored the effect of aztreonam on root induction and other related qualities after discovering its key function in soybean transformation. Aztreonam was contrasted with indole-3-butyric acid. The regenerated shoots were used for the comparison of two compounds. We employed the same concentration of aztreonam for root induction as we had for transformation. Similar IBA concentrations, meanwhile, were found to be ineffective for root induction. As a result, we raised the IBA concentration to 4 mg/L, which in our case was effective in root induction of the soybean seedlings (Figures 3A1,B1). For simultaneous root induction in soybeans, 2.5 mg/L aztreonam and 4 mg/L IBA were compared. The root induction time for both chemicals was the same; the shoot began rooting after 7 days of cultivation on RIM. However, the number of roots was not significantly different between IBA and aztreonam-treated shoots (*p* = 0.27) (Figure 3C). The root length of the seedling differed significantly (*p* = 0.002) when treated with either aztreonam or IBA. Aztreonam generated long white shoots, but root growth was slow in the IBA-containing medium. Visual examination revealed differences in root pattern, such as root color and root thickness (Figures 3A2,B2,D). According to our observation, aztreonam also greatly affected lateral root formation. A large number of lateral roots have been observed when seedlings were allowed to grow further on media containing aztreonam (data not given). The discovery of aztreonam’s unique impact on root induction could also be very beneficial during the rooting stage of transgenic development.

### 3.3. Aztreonam stimulates the accumulation of flavonoid compounds in soybean

**Figure 4:**
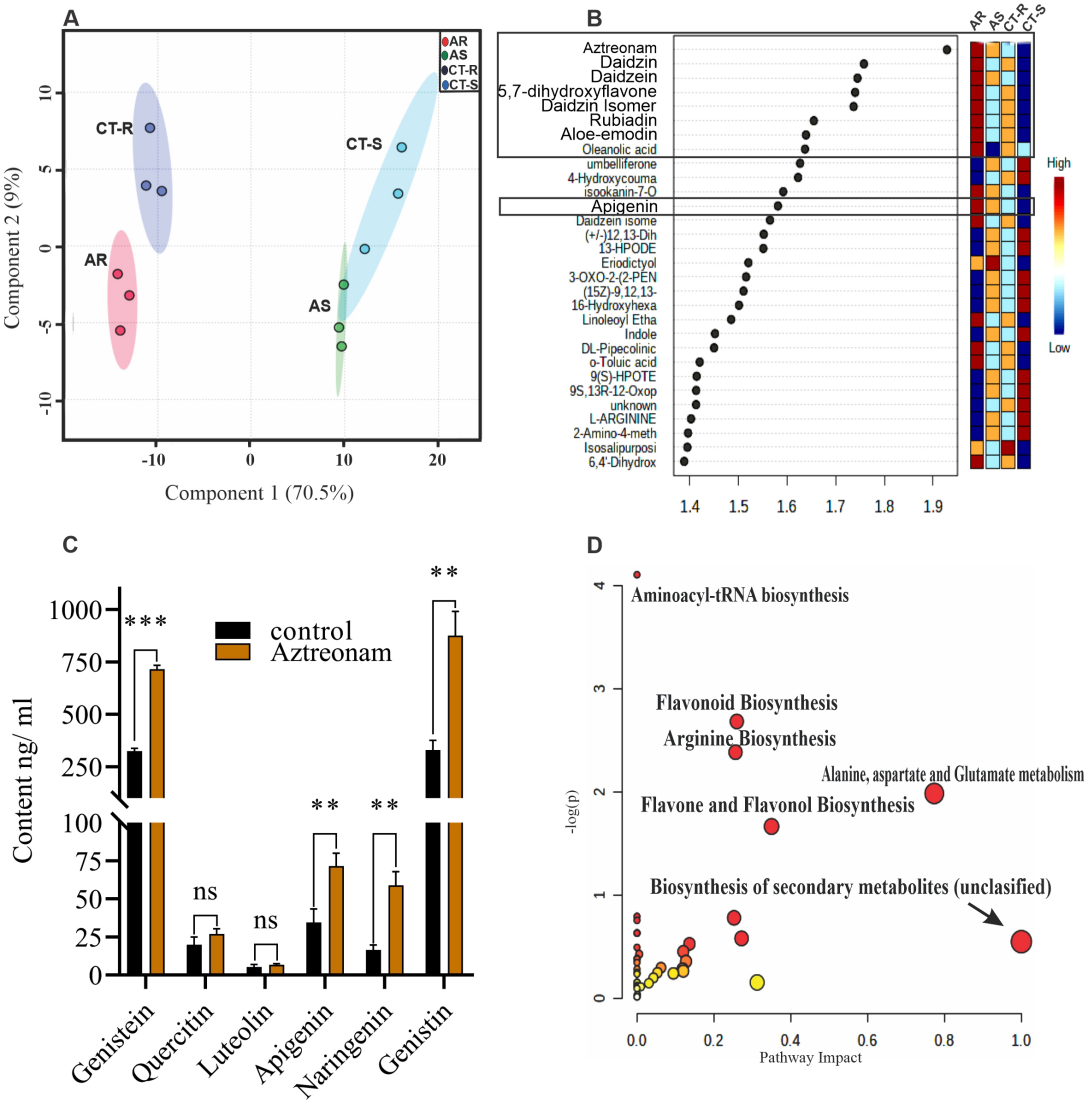
The untargeted and targeted metabolic analyses of the explants after aztreonam treatment were compared with the control. Panel **(A)** is the PLS-DA biplot, which clearly distinguishes between treated and control samples. The letters CT-R and CT-S refer to the control hypocotyl and epicotyl, respectively. However, the treated samples are represented by AS (hypocotyls) and AR (epicotyls). Panel **(B)** displays the high accumulated metabolites measured against their VIP score, which was >1. The color bars indicate the intensities of each metabolite in the sample. Graph **(C)** shows the quantification of six flavonoids, *viz.*, apigenin, genistein, naringenin, luteolin, quercetin, and genistin glycoside, in treated and control samples. Error bars show the SD of the data from the mean, while the significance of the data was analyzed by a multiple *T*-test. Significance in the data was considered when *p* was <0.005. Panel **(D)** shows the KEGG pathways (color dots represent each pathway). The x-axis displays the pathway impact, and the y-axis displays the -log10 fold value.

An untargeted metabolic study was undertaken to determine the cause of this high transformation and the beneficial effect of aztreonam on soybean root morphology. The significant increase of flavonoids in treated samples was investigated during an untargeted metabolic investigation utilizing UHPLC–MS/MS. The base peak chromatograms generated using positive and negative ionization modes revealed differences in metabolite abundance between the treatment and control samples. The majority of the flavonoid compounds were found after further sorting of the putatively detected metabolites using an online database search (Supplementary Figure S3C). Similarly, multivariate data analysis, such as heatmap metabolic patterns, reveals a large accumulation of flavone (polyphenol) group metabolites such as 2′, 6-dihydroxyflavone, 3′, 4′, 5, 7-tetrahydroxyflavone, wogonin, and liquiritigenin. In the control group, a high abundance of alkaloids has been investigated (Supplementary Figures S3A,B). As a result of the aztreonam treatment, there was a shift from alkaloid to flavonoid. The PLS-DA biplot also revealed clear divergence between the treated and control groups on components 1 and 2 (Figure 4A). Based on the analysis of differentially accumulated compounds, the majority of the flavonoids daidzein, daidzin, apigenin, and flavonol glycosides, along with rubiadin, D-glucose-6-phosphate, DL-Pipecolinic acid, and aloe-emodin, were found abundant in the treated samples (Figure 4B). The Kyoto Encyclopedia of Genes and Genomes (KEGG) revealed flavonoids as the top signaling pathway in the treated samples (Figure 4D). Flavonoid biosynthesis, arginine biosynthesis, alanine, aspartate, and glutamate metabolism, and flavone and flavonol biosynthesis pathways were identified as the top five pathways with significant *p*-values<0.05 (Supplementary Table S3). In metabolic set enrichment analysis, arginine biosynthesis was found to be significantly high, with *p* values of 0.00401 and 0.0081 (Supplementary Table S2).

### 3.4. Confirmation of high flavonoids accumulation through targeted metabolic analysis

The substantial concentration of flavonoids in soybeans following aztreonam treatment has driven further investigation of flavonoids that are specifically responsible for *Agrobacterium* virulence gene activation and bacterial chemotaxis. Five flavonoids (apigenin, genistein, luteolin, naringenin, and quercetin) were quantified in aztreonam-treated and control explants. The ethanolic extracts of the aztreonam-treated and control cotyledon explants were quantified using UHPLC-QqQ-MS/MS. The precise quantification of each compound was acquired by comparing it to known concentrations of that compound. During the quantification, one additional peak was identified as genistin, which was abundant in the treated samples. Statistical analysis showed that the accumulation of four compounds, i.e., genistein (*p* = 0.000007), apigenin (*p* = 0.0061), naringenin (*p* = 0.0015), and genistin (*p* = 0.0016), was significantly higher in cotyledon explants after aztreonam treatment (Figure 4C). However, two chemicals, quercetin and luteolin, were abundant but showed no significant differences as compared to the control.

## 4. Discussion

Several factors have been found that may help accelerate agrobacterium-mediated transformation in soybean. Among them, agrobacterium strains, vectors’ types, virulence (*vir*) gene-inducing chemicals, medium composition, and tissue specific factors are critical for boosting transformation efficiency (Opabode, 2006). The discovery of novel chemical inducers could play a crucial role in transformation. We discovered that aztreonam, a monobactam antibiotic, was extremely effective in agrobacterium-mediated transformation. No relevance for this drug in agrobacterium-mediated transformation was explored. However, other members of this group were previously reported in the tissue culture and somatic embryogenesis of different plants. Like beta-lactam antibiotics, cefotaxime, carbenicillin, penicillin G, and some other antibiotics were found effective during somatic embryogenesis, axillary bud formation, and shoot induction in *dainthus* and *Solanum viarum* (Nakano and Mii, 1993; Mahadev et al., 2014). Amoxiclav was found to increase morphogenesis and shoot regeneration in maize, carrot, and tomato, respectively (Danilova and Dolgikh, 2005; Grzebelus and Skop, 2014; Varlamova et al., 2021). However, if we utilized this during co-cultivation, its antibacterial activities against gram-negative bacteria might impede bacterial development. Initially, we utilized this antibiotic during the seed imbibition stage prior to inoculation. However, previous research findings showed that aztreonam has low antibacterial activity against *A. tumefaciens* when used during agrobacterium transformation. The minimal bactericidal concentration for *A. tumefaciens* ranged from 100 to 400 mg/L (Ogawa and Mii, 2005). Moreover, we found the lowest optimum working concentration of 2.5 mg/L to increase agrobacterium-mediated transformation. Later on, we also added this chemical during co-cultivation. The use of 2.5 mg/L during seed imbibition and then co-cultivation boosted *Agrobacterium* transformation in soybeans surprisingly.

How did aztreonam help with transformation? To address this, we investigated the metabolic divergence of treated explants. According to previous findings, ROS plays an important role against *agrobacterial* infection (Zhao et al., 2020). However, flavonoids greatly help in ROS scavenging, leading to the normal delivery of T-DNA into the plant genome (Agati et al., 2012). A substantial concentration of flavonoids and certain energy-producing chemicals were investigated during the untargeted metabolic analysis after aztreonam treatment. Flavonoids were also thought to be useful for plant-pathogen interactions and T-DNA transfer. They play an important role in quorum sensing, promoting rhizobacteria-plant interaction, and making positive changes in root microbiomes (Wang L. et al., 2022). Previously, the functional analysis of another antibiotic, sulphonamide, also resulted in enriched plant-pathogen interaction and plant hormone signal transduction pathways (Yang et al., 2020). On the other hand, the high abundance of arginine after aztreonam treatment has been observed. As arginine was previously investigated as the precursor of nopaline compounds that express in tumorous tissues (Montoya et al., 1977), some other phenolic compounds like vanillin, coumarin, and cinnamic acid were also found to be more effective as compared to acetosyringone (Cha et al., 2011). Similarly, the phenolic compound chloroxynil was the *vir* gene inducer during *Agrobacterium*-mediated transformation in *Lotus japonicas* transient expression. The transformation was six-fold higher using a lower concentration of this compound as compared to acetosyringone (Kimura et al., 2015). Our untargeted metabolic analysis did not reveal the precise profile of particular metabolites involved in agrobacterium transformation. We selected flavonoids that are primarily responsible for *Agrobacterium*-mediated transformation. Five flavonoids, i.e., genistein, apigenin, quercetin, luteolin, and naringenin, were targeted for quantification in aztreonam-treated and control soybean explants. The high concentration of these chemicals during the targeted metabolic study validated the preceding untargeted analysis. This substantial concentration also revealed that the aztreonam treatment generated flavonoids, which facilitated in the infection of the agrobacterium Zerback et al. (1989) studied the efficacy of flavonoids and their glycosides in the induction of the *VirE* locus of *A. tumefaciens.* Flavonoids like genistein, apigenin, luteolin, and naringenin were found to be the best inducers of the emrAB operon. These operons are responsible for the synthesis of bacterial cell wall inner and outer membrane proteins (Khemthong et al., 2019).

Besides transformation, aztreonam treatment affected root morphology. A large number of elongated roots were obtained after aztreonam treatment; however, no such effect was observed in IBA-treated roots. Flavonoids have also been reported in lateral root formation, nodulation, and root development. They help in auxin transport and act on HD-ZIP III and Short-root transcription factors, altering root pattern and lateral root development (Franco et al., 2015; Chapman and Muday, 2021). So, the high abundance of these metabolites implies that aztreonam treatment triggers the biosynthesis of flavonoids and opines, leading to high transformation and alteration of root morphology.

## 5. Conclusion

Due to the low transformation efficiency of soybeans, a continuous struggle is needed to increase this process for high quality and fast soybean development. The identification of novel biochemicals that either stimulate host factors that attract *A. tumefaciens* or induce pathogen virulence genes is indispensable. We demonstrated that aztreonam is responsible for triggering flavonoids in soybean tissue. We concluded that aztreonam was not directly involved in plant-pathogen interactions. However, it stimulated the essential biochemical pathways of the host plant, which induced this interaction. This high flavonoid accumulation may increase chemotaxis, reduce ROS, or activate the virulence genes of *A. tumefaciens,* leading to high-efficiency agrobacterium transformation. Further studies could explore the role of aztreonam and its effects on other metabolic regulation.

## Data availability statement

The datasets presented in this study can be found in online repositories. The names of the repository/repositories and accession number(s) can be found at this link: https://figshare.com/articles/dataset/AZTREONAM_IS_A_NOVAL_CHEMICAL_INDUCER_THAT_PROMOTES_AGROBACTEIUM_TRANSFORMATION_AND_LATERAL_ROOT_DEVELOPMENT_IN_SOYBEAN/23723724.

## Author contributions

MW: Conceptualization, Formal analysis, Methodology, Software, Writing – original draft. WY: Methodology, Writing – review & editing. KY: Writing – review & editing. XZ: Project administration, Supervision, Writing – review & editing.

## Funding

The author(s) declare financial support was received for the research, authorship, and/or publication of this article.

## Conflict of interest

The authors declare that the research was conducted in the absence of any commercial or financial relationships that could be construed as a potential conflict of interest.

## Publisher’s note

All claims expressed in this article are solely those of the authors and do not necessarily represent those of their affiliated organizations, or those of the publisher, the editors and the reviewers. Any product that may be evaluated in this article, or claim that may be made by its manufacturer, is not guaranteed or endorsed by the publisher.

## Footnotes

^1^https://pubchem.ncbi.nlm.nih.gov/

^2^https://www.mzcloud.org/
