# Supplementary material for: Aztreonam is a novel chemical inducer that promotes Agrobacteium transformation and lateral root development in soybean
Source: Front Microbiol. 2023 Aug 24;14:1257270. doi: 10.3389/fmicb.2023.1257270 (PMC10483135; doi:10.3389/fmicb.2023.1257270)
Supplement: Supplementary file 1 [file Data_Sheet_1.pdf]

Supplementary Material

AZTREONAM IS A NOVEL CHEMICAL INDUCER THAT PROMOTES AGROBACTEIUM TRANSFORMATION AND LATERAL ROOT DEVELOPMENT IN SOYBEAN

M. Waqar Khan*, Wenqi Yang, Ke Yu, Xuebin Zhang*

Corresponding author:
Xuebin Zhang*
Email: xuebinzhang@henu.edu.cn

Muhammad Waqar Khan
Email: waqar@henu.edu.cn

This file includes:

Figures S1 to S4
Tables 1 to 3

Raw HPLC data and Sanger sequence file is available in FigShare public repository: DOI.10.6084/m9.figshare.23723724

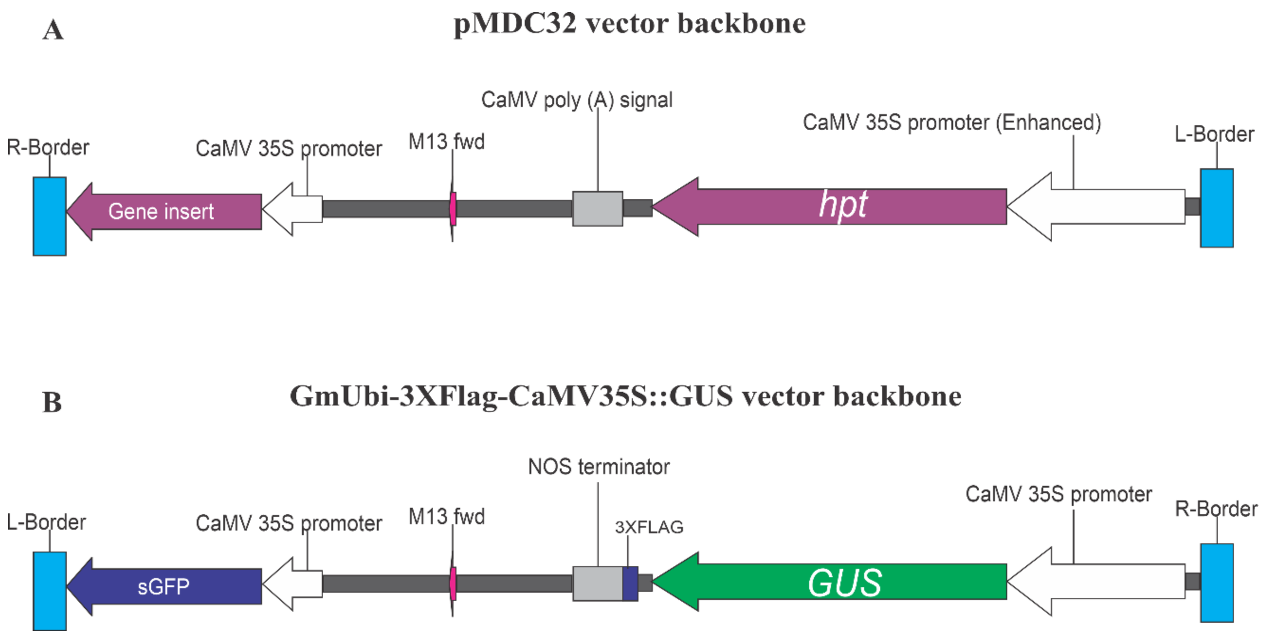

Figure S1: The desired reconstructed parts can be seen in the T-DNA sections. (A) The *hpt* gene is shown as a selectable marker for transgenic identification in the T-DNA sections of pMDC32 binary vector. B**)** A modified GmUbi-3XFlag-GFP vector was used to express GUS under the CaMV 35S promoter. This vector also contained sGFP as reporter gene driven by CaMV 35S promoter.

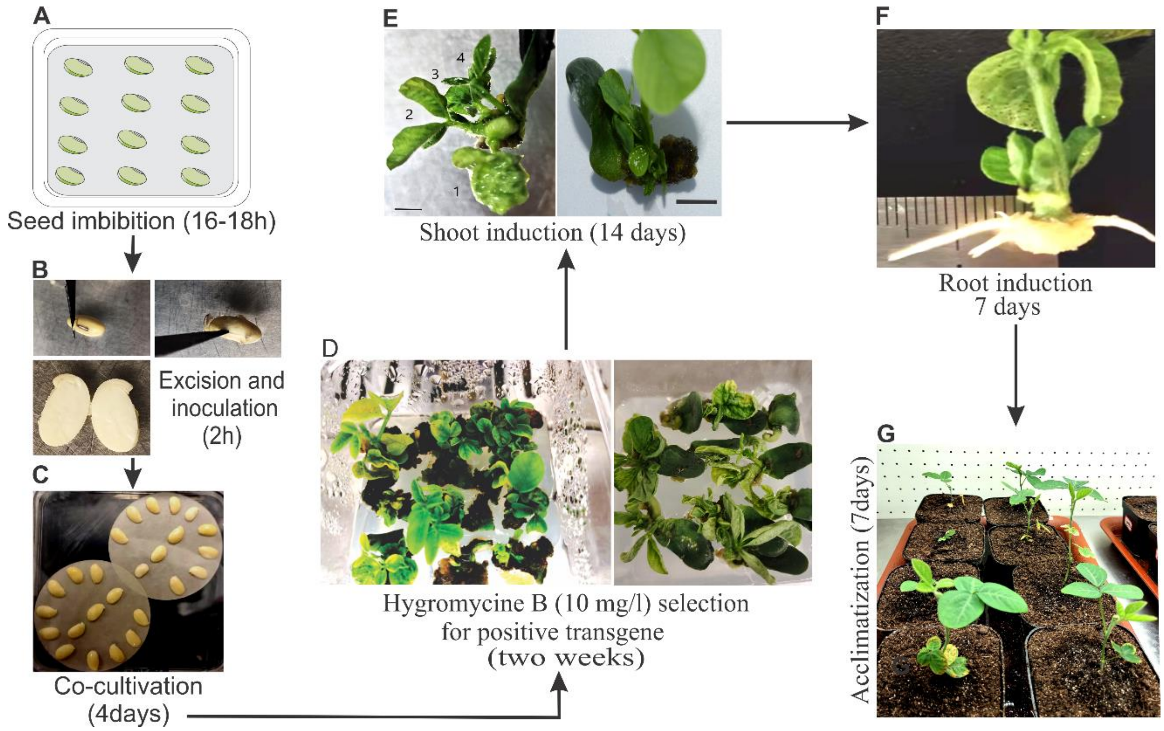

Figure S2: Soybean cotyledon inoculation and shoot regeneration steps; Different pictures represent each stage of transgene production, the pictures are alphabetically ordered from seed imbibition (A) to fully acclimatized putative transgene (F). E) Transgenes containing *hpt* gene were cultivated on media supplemented with hygromycin B prior to rooting. Total duration from seed imbibition to acclimatized seedling was 6 weeks.

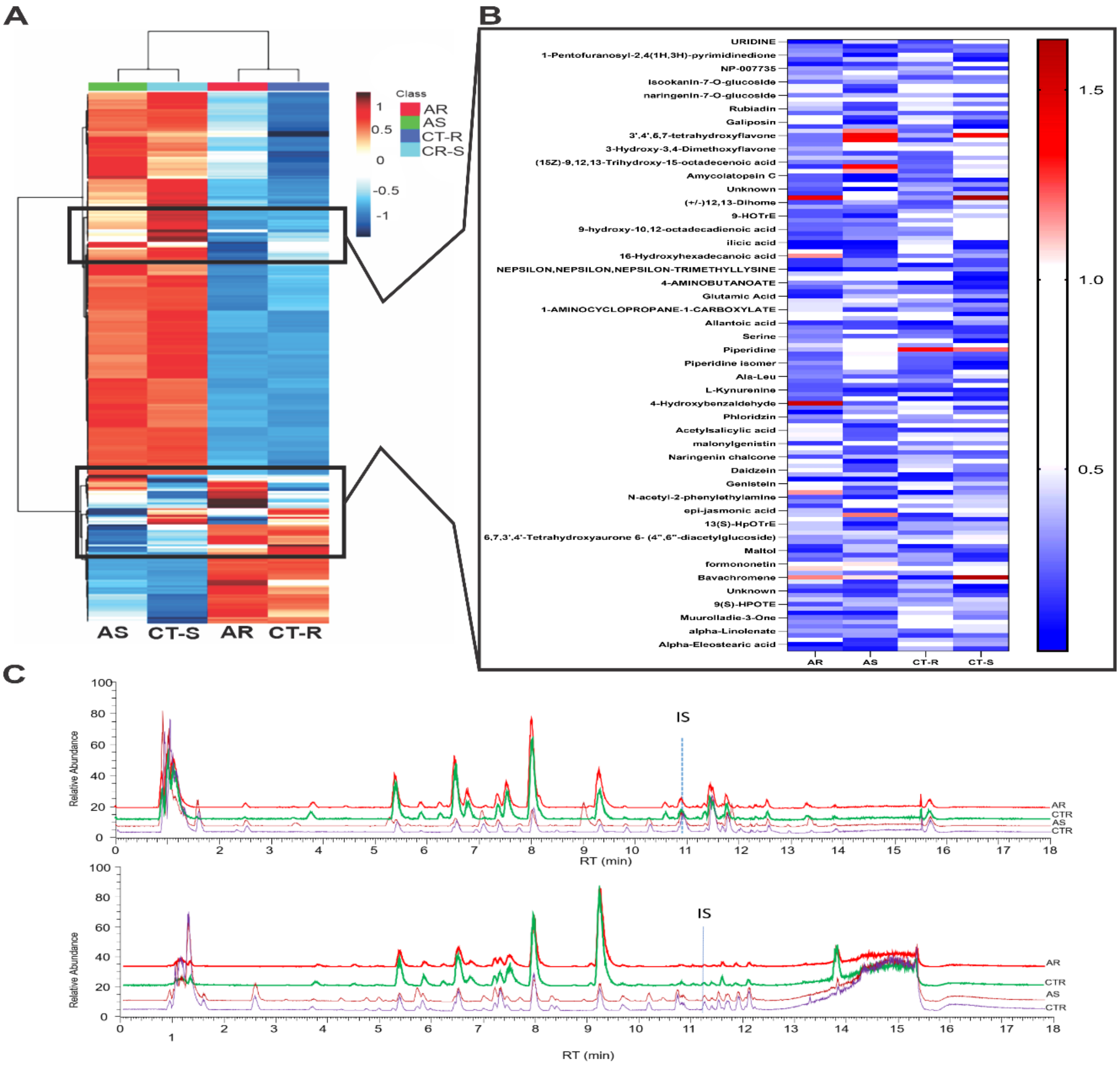

Figure S3. UHPLC-MS/MS analysis of the metabolites that were collected on positive and negative ionization modes. Metabolites from the treatment and control groups were analyzed using the MetaboAnalyst platform. **A)** A heatmap shows the variation in the absolute abundance of several metabolic features. Rectangles have been used to highlight variably accumulated metabolites in the samples, **B)** a zoomed-out view of the chosen metabolites reveals some flavonoids and a varied pattern of metabolites. **C)** The relative abundance of the metabolite in the samples is shown by the peak height in the base-peak chromatogram of the eluted metabolites.

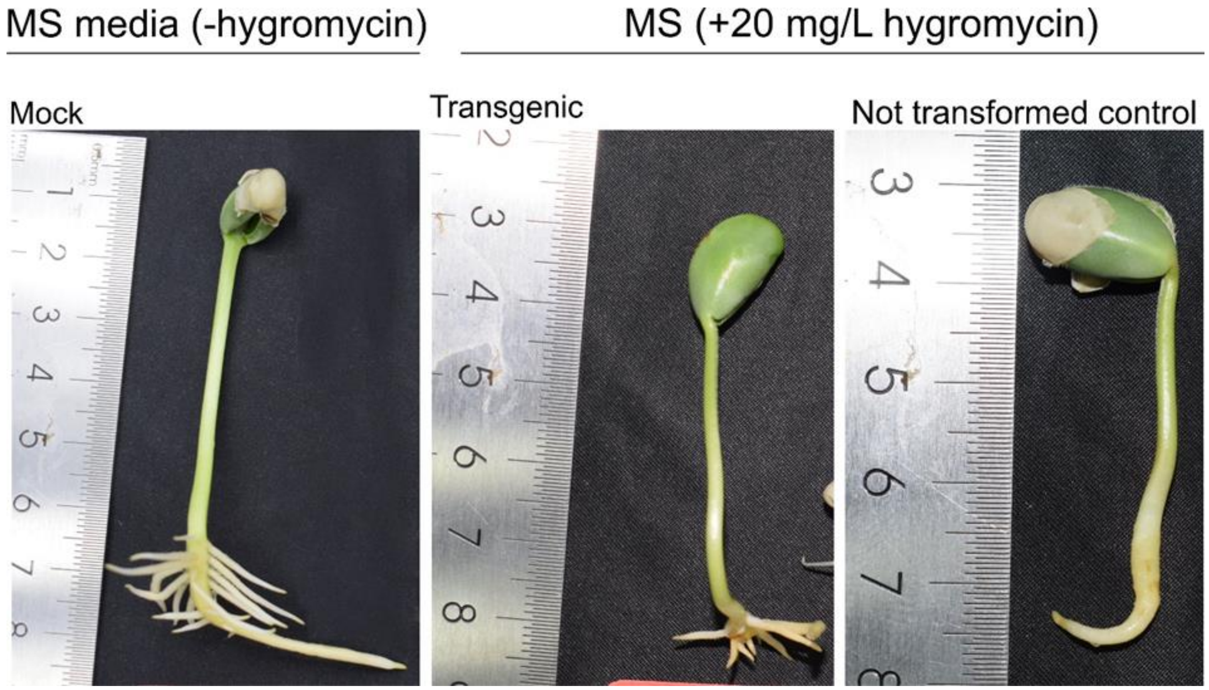

Figure S4: Assessment of positive transgenic soybean on hygromycin B selective media. Mock represent non-transgenic line grown on ¼ strength MS media without hygromycin B. Transgenic and non-transformed control were grown on ¼ strength media supplemented with 20 mg/L hygromycin B.

Table S1: List of overlapping PCR primers for GUS and CaMV 35S fragment cloning into vector

Primers Name	forward (F) Oligo	reverse (R) Oligo
CaMV Overlapping	5′- ACGTAAACCCATtctagagagata gatttgtagagagagactggtgat	5′- ccagtgccaagctgggcatggt ggagcacgacactctc
GUS-Overlapping	5′-gactcgacagtctagaATGGGTTTACG TCCTGTAGAAACC	5′-tccttatagtccatggtaccTCATTG TTTGCCTCCCTGCTGC
Positive Bacterial colonies detection primers	5′-tccttatagtccatggtaccTCATTGTTT GCCTCCCTGCTGC	5′-ccagtgccaagctgggcatggtgga gcacgacactctc

Table S2: Enriched metabolic description and their respective values.

Enriched metabolites classes	Raw p	Holm p	FDR
Arginine biosynthesis	0.00401	0.336	0.336
D-Glutamine and D-glutamate metabolism	0.00811	0.673	0.341
Riboflavin metabolism	0.0938	1	1
Aminoacyl-tRNA biosynthesis	0.108	1	1
Nitrogen metabolism	0.137	1	1
Valine, leucine and isoleucine biosynthesis	0.179	1	1
Purine metabolism	0.475	1	1
Alanine, aspartate and glutamate metabolism	0.501	1	1
Glutathione metabolism	0.501	1	1
Glyoxylate and dicarboxylate metabolism	0.549	1	1
Arginine and proline metabolism	0.612	1	1
Pyrimidine metabolism	0.622	1	1
Valine, leucine and isoleucine degradation	0.631	1	1
Tryptophan metabolism	0.64	1	1
Tyrosine metabolism	1	0.649	1

Table S3: Statistics of identified enriched pathways during untargeted metabolic profiling of aztreonam treated soybean through HPLC-MS

Identified pathways	Raw p	-log10(P)	Holm adjust	FDR	Impact
Aminoacyl-tRNA biosynthesis	7.75E-05	4.1105	0.007443	0.007443	0
Flavonoid biosynthesis	0.002051	2.688	0.19485	0.098451	0.25968
Arginine biosynthesis	0.004073	2.3901	0.38288	0.13034	0.25632
Alanine, aspartate and glutamate metabolism	0.010223	1.9904	0.9507	0.24534	0.77338
Flavone and flavonol biosynthesis	0.021366	1.6703	1	0.41023	0.35
Valine, leucine and isoleucine biosynthesis	0.15897	0.79869	1	1	0
Arginine and proline metabolism	0.1643	0.78435	1	1	0.25242
Nitrogen metabolism	0.17368	0.76026	1	1	0
Linoleic acid metabolism	0.23065	0.63705	1	1	0
Indole alkaloid biosynthesis	0.23065	0.63705	1	1	0
alpha-Linolenic acid metabolism	0.25976	0.58543	1	1	0.27203
Biosynthesis of secondary metabolites - unclassified	0.27954	0.55355	1	1	1
Butanoate metabolism	0.29359	0.53225	1	1	0.13636
beta-Alanine metabolism	0.3177	0.49799	1	1	0
Glycine, serine and threonine metabolism	0.34894	0.45725	1	1	0.1204
Purine metabolism	0.36939	0.43251	1	1	0.00569
Monobactam biosynthesis	0.40853	0.38877	1	1	0
Phenylalanine, tyrosine and tryptophan biosynthesis	0.41158	0.38554	1	1	0
Pantothenate and CoA biosynthesis	0.43412	0.36239	1	1	0.12743
Lysine biosynthesis	0.44622	0.35045	1	1	0
Glutathione metabolism	0.49887	0.30201	1	1	0.06248
Phenylalanine metabolism	0.51463	0.28851	1	1	0
Riboflavin metabolism	0.51463	0.28851	1	1	0.11852
Tryptophan metabolism	0.53937	0.26811	1	1	0.12037
Cyanoamino acid metabolism	0.55877	0.25277	1	1	0
Glyoxylate and dicarboxylate metabolism	0.55877	0.25277	1	1	0.0531
Phenylpropanoid biosynthesis	0.56778	0.24582	1	1	0.0945
Nicotinate and nicotinamide metabolism	0.57466	0.24059	1	1	0
Histidine metabolism	0.62733	0.2025	1	1	0.04264
Valine, leucine and isoleucine degradation	0.69305	0.15924	1	1	0
Lysine degradation	0.69448	0.15834	1	1	0
Cutin, suberine and wax biosynthesis	0.69448	0.15834	1	1	0.3125
Pyrimidine metabolism	0.70726	0.15042	1	1	0.03089
Carbon fixation in photosynthetic organisms	0.74963	0.12515	1	1	0

Zeatin biosynthesis	0.74963	0.12515	1	1	0
Biosynthesis of unsaturated fatty acids	0.76573	0.11593	1	1	0
Starch and sucrose metabolism	0.76573	0.11593	1	1	0.00967
Glucosinolate biosynthesis	0.79487	0.099702	1	1	0
Ubiquinone and other terpenoid-quinone biosynthesis	0.91962	0.036391	1	1	0.00097
Cysteine and methionine metabolism	0.95313	0.020846	1	1	0
Porphyrin and chlorophyll metabolism	0.95907	0.018151	1	1	0
